# Modeling the Dynamics of *Plasmodium vivax* Infection and Hypnozoite Reactivation *In Vivo*

**DOI:** 10.1371/journal.pntd.0003595

**Published:** 2015-03-17

**Authors:** Adeshina I. Adekunle, Mykola Pinkevych, Rose McGready, Christine Luxemburger, Lisa J. White, François Nosten, Deborah Cromer, Miles P. Davenport

**Affiliations:** 1 Centre for Vascular Research, UNSW Australia, Sydney, New South Wales, Australia; 2 Shoklo Malaria Research Unit, Mahidol-Oxford Tropical Medicine Research Unit, Faculty of Tropical Medicine, Mahidol University, Mae Sot, Thailand; 3 Centre for Tropical Medicine, Nuffield Department of Medicine, University of Oxford, Oxford, United Kingdom; 4 Mahidol-Oxford Tropical Medicine Research Unit, Faculty of Tropical Medicine, Mahidol University, Bangkok, Thailand; Johns Hopkins Bloomberg School of Public Health, UNITED STATES

## Abstract

The dynamics of *Plasmodium vivax* infection is characterized by reactivation of hypnozoites at varying time intervals. The relative contribution of new *P*. *vivax* infection and reactivation of dormant liver stage hypnozoites to initiation of blood stage infection is unclear. In this study, we investigate the contribution of new inoculations of *P*. *vivax* sporozoites to primary infection versus reactivation of hypnozoites by modeling the dynamics of *P*. *vivax* infection in Thailand in patients receiving treatment for either blood stage infection alone (chloroquine), or the blood and liver stages of infection (chloroquine + primaquine). In addition, we also analysed rates of infection in a study in Papua New Guinea (PNG) where patients were treated with either artesunate, or artesunate + primaquine. Our results show that up to 96% of the *P*. *vivax* infection is due to hypnozoite reactivation in individuals living in endemic areas in Thailand. Similar analysis revealed the around 70% of infections in the PNG cohort were due to hypnozoite reactivation. We show how the age of the cohort, primaquine drug failure, and seasonality may affect estimates of the ratio of primary *P*. *vivax* infection to hypnozoite reactivation. Modeling of *P*. *vivax* primary infection and hypnozoite reactivation provides important insights into infection dynamics, and suggests that 90–96% of blood stage infections arise from hypnozoite reactivation. Major differences in infection kinetics between Thailand and PNG suggest the likelihood of drug failure in PNG.

## Author Summary

*Plasmodium vivax* is one of two major parasite species causing human disease. This parasite can lie dormant in the liver as a hypnozoite, before later reactivating to cause blood-stage infection. Treatment to eliminate the dormant hypnozoite stage relies mostly on a single drug—primaquine. Understanding the rate of primary infection versus hypnozoite reactivation is important to understanding primaquine efficacy and drug resistance, as well as the development of new drugs targeting hypnozoites. Here we use mathematical modeling to analyse data from two clinical cohorts and show that up to 96% of infections may be caused by hypnozoite reactivation. We also use modeling to understand the impact of drug resistance, seasonal infection and subject age.

## Introduction

*Plasmodium vivax* is one of the major agents of malaria infection, with around 2.5 billion people living in areas at risk of infection, and more than 70 million estimated annual infections [1–3]. *P*. *vivax* is generally less pathogenic than *Plasmodium falciparum* infection due to the absence of sequestration and cytoadherence, and all blood-stage forms can be detected in peripheral circulation[4]. *P*. *vivax* also differs due to the development of dormant hypnozoite forms in the liver, which serve as reservoir of infection after the clearance or treatment of the acute blood stage of infection. *P*. *vivax* shows a preference for reticulocytes as its host cell in the blood-stage of its life-cycle [5].

The potential for reactivation of dormant hypnozoites creates a number of difficulties in understanding the transmission dynamics of *P*. *vivax* infection[6], however the majority of diagnosed infections are thought to be due to hypnozoite reactivation rather than new primary infection [7–9]. After treatment to eliminate blood stage infection, new infections may occur as a result of recrudescence (failure of treatment of the blood stage), hypnozoite reactivation, or new primary infection. Although comparison of *P*. *vivax* genotypes may be useful in distinguishing recrudescence of the blood stage parasites after treatment, it is not always useful in differentiating reactivation of a dormant hypnozoite from new primary infection [10–12]. This is because the parasites causing relapses are often genetically different from those observed in the most recent blood-stage infection, so it is not possible to differentiate reactivation from new primary infection using genotyping[13, 14]. Therefore, it is difficult to know the proportion of blood stage infections due to hypnozoite reactivation versus new primary infection by *P*. *vivax*.

In this study, we aim to apply mathematical modeling to quantify the relative contribution of new primary infection, and infection initiated due to the reactivation of dormant liver stage hypnozoites. We analyse data from two published prospective studies where individuals were treated to eliminate blood-stage infection, and a subset were treated with primaquine, a licensed radical treatment for hypnozoites, to eliminate preexisting hypnozoites [15, 16]. By comparing the rate of observed blood stage infection in the two groups, we can estimate the contribution of primary infection versus hypnozoite reactivation in *P*. *vivax* infection.

## Materials and Methods

### Thailand study data

The field study data were from a published prospective study with recruitment from July 1995 to July 1996, on 342 individuals of different ages (68% (258) <15 years of age) living on the western border of Thailand where *P*. *vivax* is endemic[9]. Individuals with asexual forms of *P*. *vivax* on a blood smear were enrolled, treated with chloroquine (25mg base/kg over 3 days) and followed up until presentation of pure or mixed *P*. *vivax* blood stage infection. Individuals with reappearance of pure *P*. *vivax* infection were retreated with either chloroquine only (70 individuals with mean age (range) 12 (1–50)) or chloroquine and primaquine (0.25 mg/kg daily for 14 days, 43 individuals with mean age (range) 13 (5–43)), and were followed up by microscopy until detection of *P*. *vivax* blood stage parasites. Each dose of chloroquine was supervised and the patient observed for 1 hour after dosing. The criteria for enrollment and method of detection and quantification of *P*. *vivax* parasites in the blood smears are detailed elsewhere[9]. From the fact that primaquine can kill liver stage hypnozoites[15, 16] we classified the data in two groups: individuals retreated with chloroquine+primaquine (CQ+PQ) group and individuals retreated with chloroquine only (CQ only) group.

### Papua New Guinea (PNG) study

We also analysed published data on a treatment-time-to-infection study in PNG [8]. In this study, the contribution of relapse to the risk *P*. *vivax* infection and disease was studied in 433 PNG children 1–5 years of age. Children were randomized into one of three groups: (1) artesunate (4mg/kg/d for 7 days) plus primaquine (0.5 mg/kg/d for 14 days), 149 individuals, (2) artesunate only (4 mg/kg/d for 7 days), 150 individuals or (3) no treatment (control), 150 individuals, and were followed up for infection and the presence of febrile illness. Every dose of treatment was administered as direct observed therapy. The criteria for enrollment, method of randomization, treatment procedures and method for the detection and quantification of *P*. *vivax* parasites in the blood smears were detailed in the original publication [8]. For our analysis we extracted data on infection rates from Fig. 2 of Betuela et al [8], using Grafula 3.0 (Knowledge Probe Inc, Aurora)to extract data on cumulative proportion of infections detected by light microscopy. The data was classified into two groups; those receiving artesunate+primaquine (AS+PQ group), and artesunate only (AS only) group).

### Modeling dynamics of *P*. *vivax* infection

The dynamics of *P*. *vivax* infection are characterized by both primary infection and reactivation. For individuals living in *P*. *vivax* endemic regions both primary infection and activation of hypnozoites occur throughout the year. For modelling purposes we will label the two groups of subjects as i) the B+H group, comprising individuals who received drugs against both blood stage parasites and hypnozoites and ii) the B group, comprising individuals who received drugs against blood stage parasites only. We initially assume 100% drug efficacy against both the blood and liver stage parasites. Thus for individuals receiving in the B+H group, all observed infection is due to new primary infection. For individuals in the B group, infections can arise either from new primary infection, or from reactivation of hypnozoites. Thus, we can model the time to first *P*. *vivax* infection after treatment, and fit this to the ‘survival curve’ of time to detection of *P*. *vivax* infection after treatment. The proportion of treated individuals in the B+H group remaining uninfected at a given time *t* is indicated as (*S*_*B+H*_(*t*)) and follows the equation:

$$S_{B+H}\left(t\right)=\left\{ \begin{array}{ll} 1, & t\leq d_{1}\\ e^{-k(t-d_{1})}, & t>d_{1} \end{array},\right.$$ (1)

and the proportion of treated individuals in the B group remaining uninfected at time *t* is indicated as (*S*_*B*_ *(t)*) and follows the equation

$$S_{B}\left(t\right)=\left\{ \begin{array}{ll} 1, & t\leq d_{2}\\ e^{-k(1+c)\left(t-d_{2}\right)}, & t>d_{2} \end{array}\right.$$ (2)

**Fig 1:**
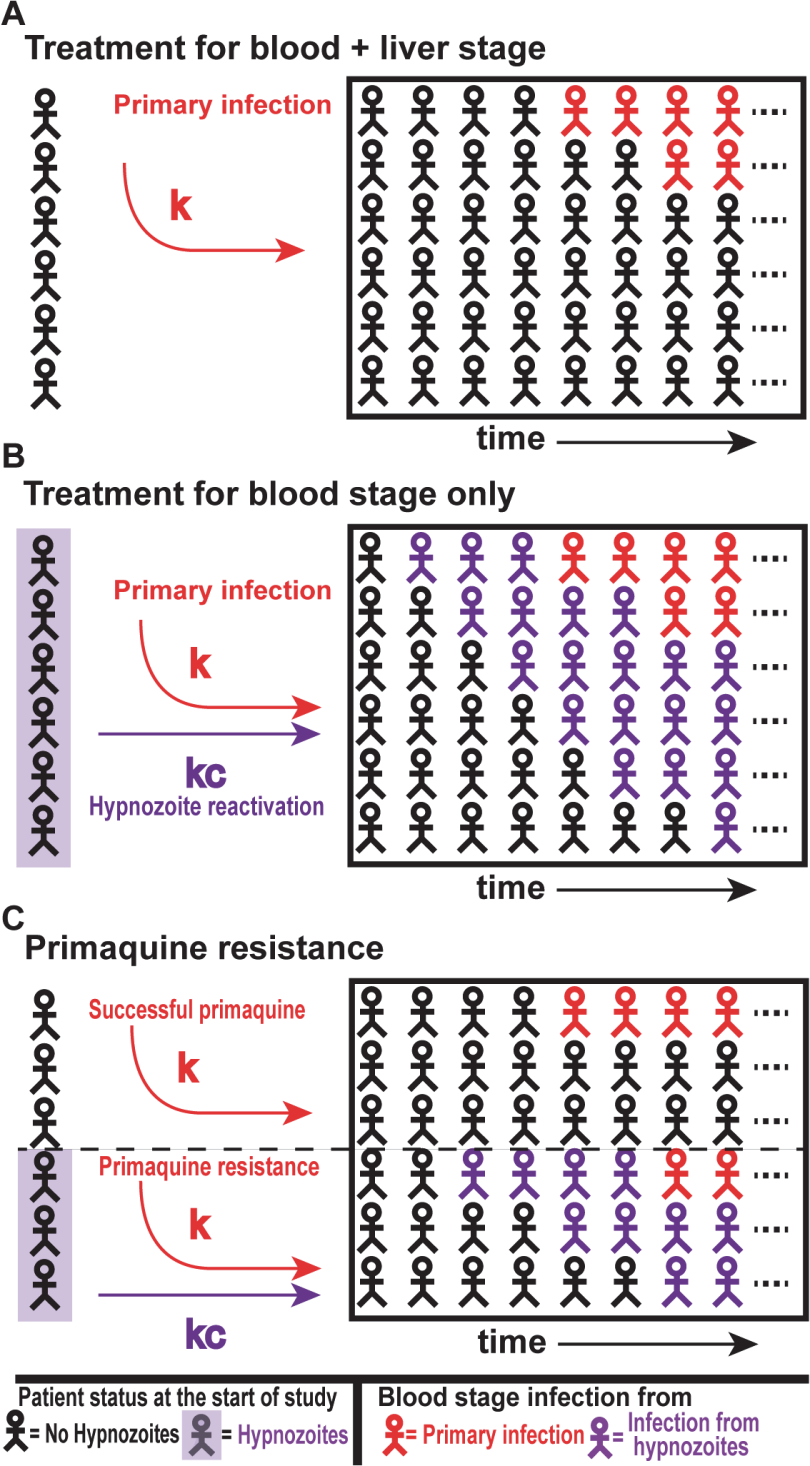
Schematic of primaquine treated *P*.*vivax* infection in the study population. (A) shows the effect of successful liver and blood stage treatment of individuals in a *P*.*vivax* endemic region over time. Individuals become infected due to primary infection at the rate *k*, as the hypnozoite reservoir has been successfully cleared. In panel B, individuals are treated for blood stage parasites only. This results in blood stage infection from hypnozoite reactivation at the rate *kc*, and new infectious mosquito bites at the rate *k*. Panel C shows a scenario where hypnozoites and blood stage parasites are successfully cleared in some proportion of the population (top half of panel), and this population experiences new primary infections only (at rate *k*). The remaining proportion of the population has primaquine resistance, and thus retains their reservoir of hypnozoites. These resistant individuals will become infected at the rate *k* for primary infection and *kc* for hypnozoite reactivation.

Here *k* is the rate of initiation of new primary infections, *c* is the relapse to reinfection ratio and *d*_1_ and *d*_2_ are delays to detection of blood stage *P*. *vivax* parasites in the groups respectively. Fig. 1A and 1B show the schematic representation of Equation (1) and (2) where individual received treatments for either both blood stage and liver stage parasites or blood stage parasites only.

### Modeling dynamics of *P*. *vivax* infection with primaquine resistance

The modeling above assumes that all primaquine is completely effective in all individuals. However, if primaquine fails to kill hypnozoites from a proportion of strains, or in a proportion of individuals, then we expect altered dynamics. If primaquine kills only a fraction of hypnozoites, then the equation for *S*_*B+H*_(*t*) becomes:

$$s_{B+H}\left(t\right)=\left\{ \begin{array}{ll} 1, & t\leq d_{1}\\ e^{-k(1+Rc)\left(t-d_{2}\right)}, & t>d_{1} \end{array}\right.$$ (3)

Where *R* is the fraction of hypnozoites that are resistant to primaquine. We note that in our study we cannot differentiate between this and Equation (2) with a different *c*.

If primaquine only kills hypnozoites in a subset of individuals, then primaquine resistant individuals will behave as if they have not received primaquine. Thus, if primaquine is only effective in a proportion of individuals, the rate of infection of individuals in the B+H group will be;

$$s_{B+H}\left(t\right)=\left\{ \begin{array}{ll} 1, & t\leq d_{1}\\ {Pe}^{-k\left(t-d_{1}\right)}+(1-P)e^{-k(1+c)\left(t-d_{1}\right)}, & t>d_{1} \end{array},\right.$$ (4)

where *P* is the proportion of individuals in whom primaquine is effective. The dynamics is illustrated in Fig. 1C. Equations (1), (2) and (4) were fit to the data using the *lsqnonlin* function in MATLAB R2012 (Release M (2012) The MathWorks Inc, Natick, MA, USA), which uses a nonlinear least-squares method, to understand the contribution of hypnozoites reactivation to the dynamics of *P*. *vivax* infection.

### Modeling the dynamics of *P*.*vivax* hypnozoite reactivation

To understand the different contributions of primary infection and reactivation, we need to understand how the force of infection from primary infection and hypnozoite reactivation evolve over time / with exposure starting with a naïve host. If we consider a fixed rate of infectious mosquito inoculation (*M*), and that each inoculation infects a number of liver cells (*S*) that will eventually go on to produce a blood stage infection, then the total rate of successful infection of liver cells is simply *MS*. Assuming that the time spent in the liver stage is negligible for primary infection, the rate of new primary infections (*I*_*p*_) is simply the overall rate of infection of liver cells *MS* multiplied by the fraction of liver cell infections that result in primary infection (*f*):

$$I_{P}=fMS$$ (5)

We note that *fMS* is equivalent to *k* in Equations 1–4. Importantly, the rate of successful infection of liver cells may not be the same as the rate of successful mosquito inoculation (as one successful inoculation may infect one or more liver cells, see Fig. 1). Moreover, the definition of successful infection of a liver cell is not simply that a cell becomes infected, but that the infected cell gives rise to a blood stage infection at some stage (either immediately or with some delay).

If we consider an individual that starts with no hypnozoites (for example a neonate, or someone successfully treated with primaquine), the number of hypnozoites and rate of infection from hypnozoite reactivation (*I*_*H*_) evolves over time as hypnozoites accumulate upon repeated infection. Hypnozoites accumulate dependent on the rate of infection of liver cells (*MS*), and the fraction of liver cells that become hypnozoites (*1-f*). The dynamical equation for the number of hypnozoites (*H*) over time is

$$\frac{dH}{dt}=\left(1-f\right)MS-aH$$ (6)

where *a* is the reactivation rate of hypnozoites. We note that this assumes that hypnozoites never infect more than a tiny proportion of total liver cells. The rate of infection due to hypnozoite reactivation is then simply:

$$I_{H}=aH$$ (7)

**Fig 2:**
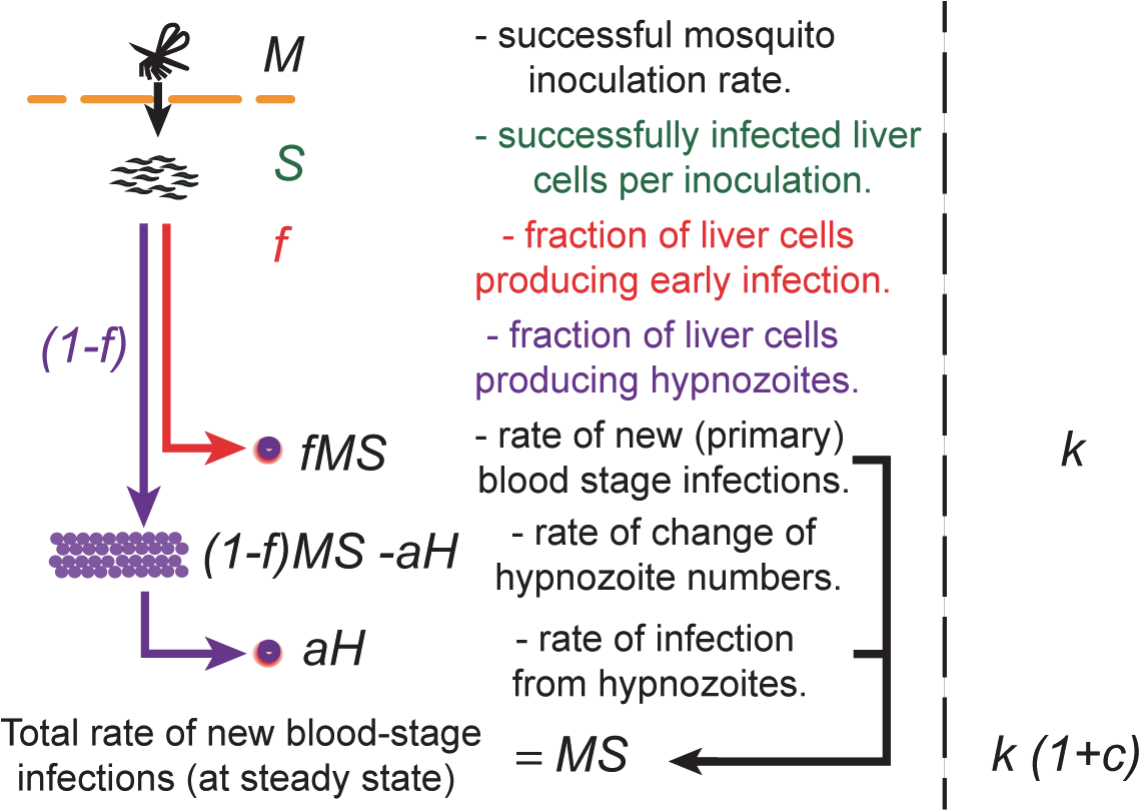
Schematic of *P*. *vivax* infection. The dynamics of *P*. *vivax* infection starts from a successful mosquito inoculation. We denote the rate of successful mosquito inoculation as *M*. Each successful mosquito inoculation successfully infects on average *S* liver cells (ie: leads to *S* blood stage infections). A fraction *f* of infected liver cells proceeds to early infection, leaving a fraction *(1-f)* as hypnozoites to later reactivate. The rate of new blood stage infections is thus *MS*, which is equivalent to rate of initiation of new infection observed in individuals treated with either chloroquine plus primaquine, or artesunate plus primaquine (*k* in Equations 1–4). Hypnozoites (*H*) are formed at a rate *(1-f)MS* and reactivate at rate *aH*. The total rate of blood stage infection (from new infection plus hypnozoites) settles to *MS* (ie: the rate of blood stage infection is the same as the rate of successful infection of new liver cells in steady state). The total rate of new blood stage infection *MS* is equivalent to the infection rate observed in individuals treated with chloroquine or artesunate alone ((*k*(1+*c*)) in Equations 1–4).

The solution of Equations (6) and (7) is

$$I_{H}=\left(1-f\right)MS\left(1-e^{-at}\right)+aH_{0}e^{-at}$$ (8)

where *H*_0_ is the initial number of hypnozoites. At steady state, the rate of infection from hypnozoites (*I*_*H*_) in Equations 7 and 8 is equivalent to *kc* in Equations 2 and 4. Similarly, the total rate of new blood stage infections at steady state is simply *MS* (the rate of successful infection of liver cells). Fig. 2 illustrates the mechanisms of infection and the relationship between parameters in Equations 1–4, and 5–7.

### Modeling the effects of seasonality

*Plasmodium vivax* infection often follows a seasonal pattern, with higher infection in the wet season[17, 18]. To understand the role of fluctuations in the force of infection and the effect of seasonality, we modified Equation (6) by allowing the rate of infectious mosquito inoculation to vary seasonally;

$$M=M_{s}\left(1+M_{f}cos(2\pi t/365)\right)$$ (9)

where *M*_*s*_ is the mean rate of infectious mosquito inoculation and *M*_*f*_ is a parameter for the degree of seasonal fluctuation. The periodicities of these functions are such they divide the season into dry and wet seasons. We examine the effect of seasonality and EIR on the contribution of primary infection to reactivation by numerical simulations of Equations (5) and (6) with seasonality terms included.

## Results

### The ratio of primary *P*.*vivax* infection to hypnozoite reactivation in Thailand

**Fig 3:**
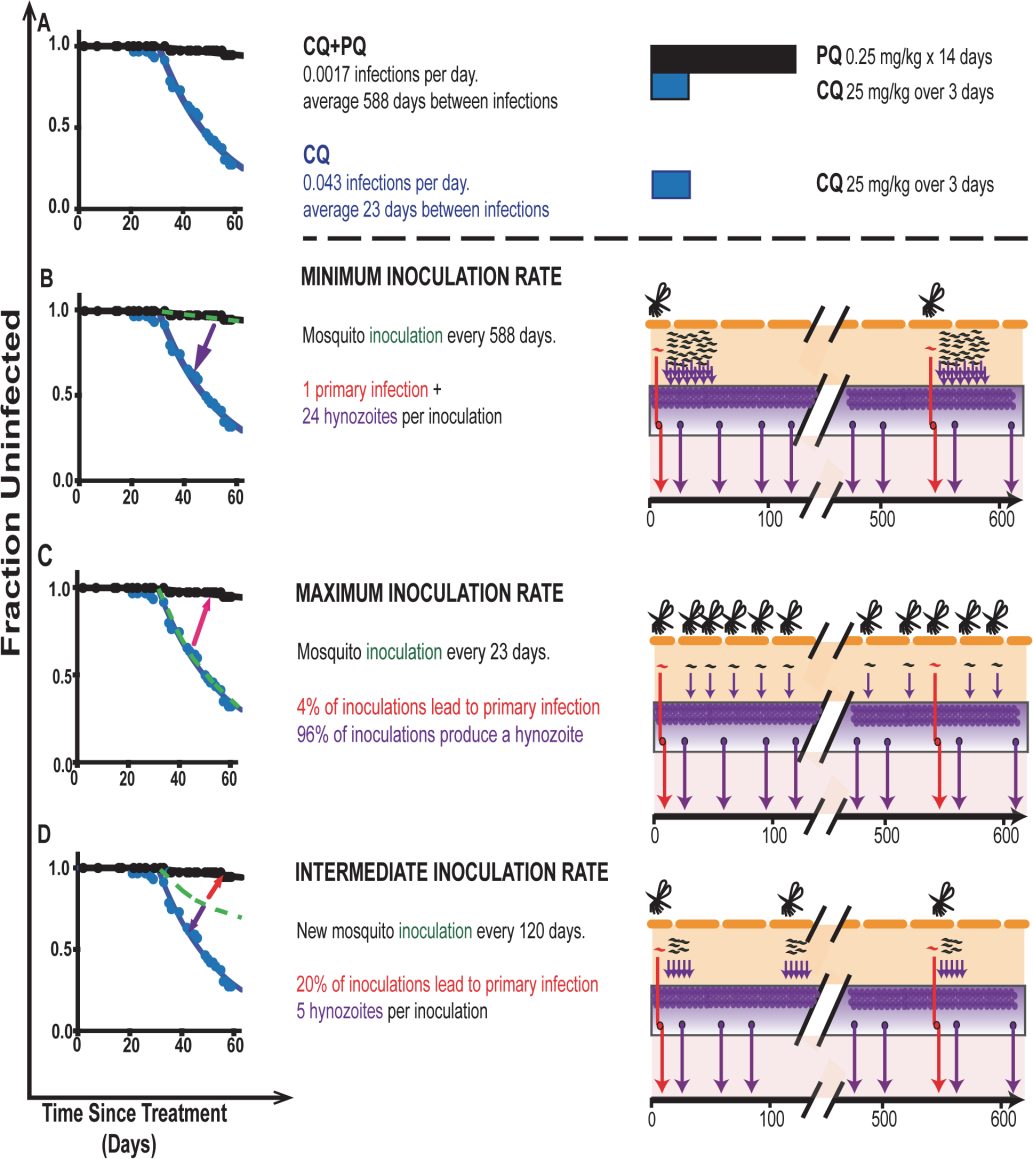
The rate of primary infection versus hypnozoite reactivation in Thailand. (A) Modelling was performed to estimate the rate of blood stage infection in individuals treated with chloroquine (blue), or chloroquine + Primaquine (black). The actual rate of inoculation of new infections cannot be directly estimated from this data. Panels B-D show several possible rates if infectious inoculation (green dashed line) that could explain the data. (B), The minimal rate of inoculation occurs if every new inoculation were observed as a primary infection. In this case, the infectious inoculation rate equals the rate of infection in the CQ+PQ group (a new inoculation every 588 days), and each inoculation must lay down 24 hypnozoites that later reactivate. (C), The maximal rate of infectious inoculation would be if each inoculation lead to either one primary infection, or one later reactivation. In this case, only 4% of inoculations would present as primary infections, and the rest lay down one hypnozoites that later reactivates. (D), The most likely infectious inoculation rate appears an intermediate between these extremes (of (B) and (C)), in which only a portion of inoculations leads to primary infection, and each inoculation lays down several hypnozoites. In panel D the green line illustrates a rate at infectious inoculation where ≈20% of inoculations leads to primary infection, and each inoculation lays down ≈5 hypnozoites.

We first estimated using Equation (1) the rate of infection in individuals receiving chloroquine plus primaquine treatment in Thailand, in whom all infections are assumed to be due to new primary infection. Assuming a constant force of infection, we found that a rate of infection of approximately 0.0017 per day (equating to an average time to primary infection of 588 days) provided the best fit to the data (Fig. 3A). We next estimated the rate of infection and reactivation occurring in the individuals that received chloroquine alone using Equation (2), where infection can arise due to both new primary infection as well as hypnozoite reactivation. In this case, we observed the rate of 0.043 infections per day (equivalent to an average of 23 days to infection). Since the individuals receiving chloroquine alone experience both primary infection and reactivation from hypnozoites, we can subtract the rate of primary infection (estimated in the CQ + PQ group) to estimate the rate of hypnozoite reactivation. Thus, we estimate a rate of hypnozoite reactivation of 0.0413 per day (equivalent to a hypnozoite reactivation every 24 days). There was no evidence for a difference in the delays from treatment to first detection in the CQ+PQ group and the CQ group (*p* = 0.8563, F-test).

The relative rates of new primary infection and hypnozoite reactivation estimated in this data suggest that approximately 4% of infection events in individuals receiving chloroquine occurred due to primary infection, and approximately 96% of infection events occurred due to hypnozoite reactivation. This implies a ratio of primary infection to hypnozoite reactivation of approximately 1 to 24.

### Hynozoite reactivation reflects hypnozoite production rate

Once hypnozoites are laid down, they will reactivate at some later time either spontaneously, or following some form of stimulation (such as fever or concurrent infection). So, for a single bite we might imagine there is some average time to reactivation, and a distribution in the probability of reactivation with time. The delay between initial inoculation and subsequent hypnozoite reactivation is highly variable[19, 20]. However, even in the absence of knowing the precise schedule of reactivation of individual hypnozoites, we can still understand the dynamics of reactivation in an endemic setting. That is, in an endemic setting we do not observe reactivation from a single inoculation, but in fact from a long series of past inoculations. We have a probability of reactivation from an inoculation 6 months ago, which is dependent on the rate of inoculation six months ago, the proportion of hypnozoites surviving 6 months, and the rate of reactivation of 6 month old hypnozoites. The same is true for hypnozoites inoculated a month ago, or a year ago. In this circumstance if we have a constant rate of inoculation for individuals of a particular age group who have been exposed to *Pv* infections for a sufficient time to reach ‘steady state’ of infection, where each the average rate of reactivation of hypnozoites reflects the rate at which they were laid down (from Equation (6), when the number of hypnozoites is constant $(\frac{dH}{dt}=0)$, then the rate of hypnozoite reactivation (*aH*) equals the rate of new hypnozoite infection *((1-f)MS*). Since treatment for blood-stage infection is relatively short-lived compared to the history of infection (and has no impact on accumulated liver stages), this should not significantly affect this steady state.

### Understanding the rate of inoculation

Although we can estimate the ratio between primary infections and reactivations, we cannot directly estimate the ‘rate of infection’ (rate of new infectious inoculation from mosquito bites) from this data. That is, for an inoculation to be infectious, it must eventually produce either primary infection, or a hypnozoite that later reactivates. It is not clear that all inoculations must produce a primary infection (some may produce only hypnozoites). Thus, the rate of new primary infection in CQ+PQ individuals may or may not reflect infectious inoculation rate, since some fraction of inoculations may produce only hypnozoites. However, the minimal rate of infectious inoculation would occur when every new inoculation produced a primary infection. If we assume that every new inoculation must result in an early, acute blood stage infection, then the minimal inoculation rate is simply the rate of new blood stage infection in the CQ + PQ group (and both inoculation and new primary infection would be experienced approximately every 588 days). If this were the case, it would also require that each new inoculation must lay down approximately 24 hypnozoites (in order to account for the observed high rate of hypnozoite reactivation in the CQ group)(Fig. 3B).

The alternative scenario occurs if some infectious inoculations do not produce a primary infection, and instead only lay down hypnozoites. Since to be an ‘infectious inoculation’ the inoculation must produce either one primary infection or one hypnozoite, the highest rate of infectious inoculation would be when each infection only produced exactly one infected liver cell, which could either produce one primary infection or one hypnozoite. In this case, the rate of infection observed in the CQ group is exactly the rate of infectious inoculation. If this were the case, then it would require that only 4% of infectious inocula presented as a primary infection, and the rest became dormant (laid down their one hypnozoite) without being observed as a primary infection (Fig. 3C). This seems unlikely, as there is experimental evidence that more than one reactivation event (and thus more than one hypnozoite) can arise from a single inoculation[21].

The analysis above describes the maximal and minimal rates of infectious inoculation, which imply very different numbers of liver cells infected per inoculation. The maximal rate suggests that 24 liver cells are infected from each infectious bite, but that this only occurs every 588 days. The minimal rate suggests that each infectious bite infects at most one liver cell, but this happens as frequently as every 23 days. The rate of infection could also be considered in terms of the rate of infection of liver cells (either as primary infection or hynozoites), even without knowing how many liver cells are infected per mosquito inoculation. The infection rate in the CQ treated group is driven by both the rate of primary infection plus the rate of hypnozoite reactivation. As discussed above, the rate of reactivation reflects the sum of all previous inoculations (at all times) and their probability of reactivating, and is thus reflective of the rate of ‘laying down’ hypnozoites. The infection rate of the CQ group is thus the total rate of infection of liver cells (either destined for primary infection or hypnozoites), assuming that only one cell initiates each primary infection or reactivation (assuming chloroquine is effective). Thus, the minimum rate of liver cell infection can be derived from the CQ group, and is 0.043 infected cells per day (a new infected liver cell every 23 days). However, unless we know the number of liver cells infected on each inoculation, or the proportion of inoculations that cause primary infection, we cannot directly estimate the inoculation rate.

From the analysis above we can estimate the minimal and maximal inoculation rates, and the minimum rate of production of new infected liver cells (which is the same as the maximal inoculation rate). Fig. 3D illustrates one of many possible scenarios in between these extremes. For example, if we had an infection rate of 0.0086 per day (equating to a new infection being established every 120 days), this would require that approximately 20% of infection were observed as a primary infection, and each infection produced on average five hypnozoites (Fig. 3D). However, it is clear that although we can estimate primary infection and reactivation rates, we cannot directly estimate the rate of infectious inoculation from the data unless we assume that each infection event always produced an observed primary infection. In addition, we cannot estimate the rate of infection of liver cells unless we assume that each infection arises from a single infected liver cell.

### Different *P*. *vivax* infection kinetics in Papua New Guinea

**Fig 4:**
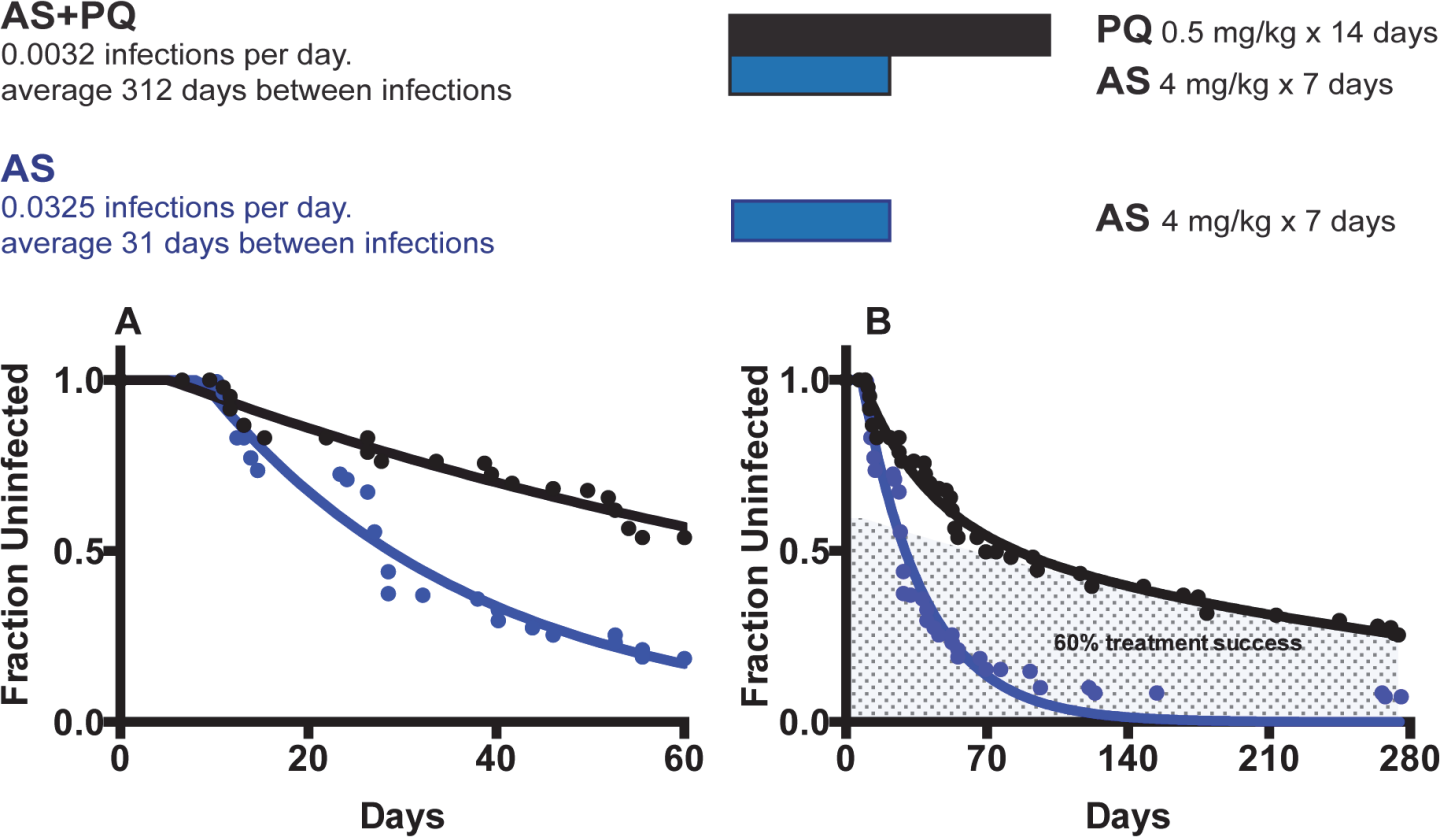
Primaquine therapy in PNG. Fitting of the mathematical model to data from PNG. (A) The models fit (solid lines) assuming 100% efficacy of primaquine treatment over the first 60 days after treatment is shown at left. The black line indicates the fit for the AS+PQ group, and the blue line for the AS only group. (B) The best fit model for the full 280 day time course is shown at right, assuming that primaquine is ineffective in a proportion of individuals (B).

In order to compare these dynamics in another population, we investigated the infection rates of patients treated with either artesunate alone, or artesunate + primaquine by analyzing a published data set from Papua New Guinea. In order to make our analysis directly comparable with the Thai study, we first restricted our analysis to infections detected by microscopy in the first 60 days since treatment (Fig. 4A). In individuals receiving artesunate plus primaquine, we observed using Equation (1) a rate of blood stage infection of 0.0102 / day (equivalent to 98 days between new blood stage infections). In individuals receiving artesunate alone, we observed using Equation (2) a rate of blood stage infection of 0.0344 / day (equivalent to a new infection every 29 days). Using the same approach as discussed above, we would estimate that in individuals treated with artesunate alone, 30% of infections occur due to primary infection and 70% due to hypnozoites reactivation (a ratio of 2.37 to 1). This ratio of reactivation to primary infection is 10 fold lower than the ratio observed in the Thai study. There was no evidence for a significant difference in the estimated delays to first detection of infection in AS+PQ group and AS only groups (*p* = 0.0807, F-test). The large difference in the ratio of primary infection to hypnozoite reactivation between the Thai and PNG studies raises a number of questions, which we explore below.

### Modeling the impact of drug efficacy on infection

One reason for the difference between the Thai and PNG studies could arise from primaquine resistance. That is, either particular strains of parasites or particular individuals may be resistant to primaquine. If primaquine were only effective in a subset of parasite strains, then only a fraction of hypnozoites (from sensitive strains) would be killed by treatment. Hypnozoite reactivation would then be reduced by this fraction, and we would see simply an increase in the (exponential) rate of infection in the primaquine treated group. Alternatively, if primaquine was only effective in a proportion of individuals, then we might expect to see a rapid rate of infection in primaquine-resistant individuals (at the same rate as those receiving artesunate alone, due to both primary infection and hypnozoite reactivation), and a slow rate of primary infection in those in whom primaquine was effective. This would produce a different (non-exponential) infection curve, characterized by two populations and two infection rates.

In order to assess whether primaquine resistance might either affect a proportion of strains, or a proportion of individuals, we modeled the full time course of infection in the PNG data (comparing Equations (1), (2) and (4)). We found that a model in which a proportion of individuals are primaquine-resistant (Equation 4) provides a significantly better fit to the data (p<0.0001, F-test). In the case of primaquine resistant individuals, the infection rate in those in whom primaquine was ineffective should be the same as the infection rate in those receiving artesunate alone, and we can estimate the rate for those with successful primaquine therapy independently. When we fit the data to this function (Equation 4), we can estimate that the best fit to the data occurs if primaquine is effective in ≈60% percent of individuals, and the rate of primary infection (in the group in which primaquine was effective) was 0.0032 / day (equivalent to a new primary infection every 313 days). Interestingly, this gives a ratio of primary infection to reactivation of 1 to 9, which is much more similar to the rate estimated from the Thai study. Table 1 shows the best-fit estimates of the models (1), (2) and (4) to the both Thai and PNG data sets.

**Table 1:** The best-fit parameters estimate for the contribution of new infection to dormant-liver stage activation.

**Thai Data**		
**Parameters**	**Infection rate (blood stage infections/day) (95% CI)**	**Average time between infections (Days) (95% CI)**
**Infection rate in CQ+PQ treated group (*k*). (Equation 1)**	0.0017 (0.0006, 0.0027)	588 (370, 806)
**Infection rate in CQ treated group (*k(1+c*)). (Equation 2)**	0.0430 (0.0163, 0.0697)	23 (14, 32)
**Ratio of relapse to reinfection (*c*)**	24.3 (8.56, 40.0)	
**% infections due to hypnozoite reactivation.**	96% (90%, 98%)	
**PNG Data (assuming 100% drug efficacy, first 60 days)**		
**Infection rate in AS+PQ group (*k*). (Equation 1)**	0.0102 (0.0085, 0.0120)	98 (83,113)
**Infection rate in AS group (*k(1+c*)). (Equation 2)**	0.0344 (0.0275, 0.0413)	29 (24,34)
**Ratio of relapse to reinfection (*c*)**	2.37 (1.69, 3.05)	
**% infections due to hypnozoite reactivation.**	70.3% (63%, 75%)	
**PNG Data (Estimating drug resistance, full Time Course)**		
**Infection rate in AS+PQ group (Equation 4).**	0.0032 (0.0023, 0.0041)	312 (243, 381)
**Infection rate in AS group (*k(1+c*)). (Equation 2)**	0.0325 (0.0232, 0.0418)	31 (24,38)
**Ratio of relapse to reinfection (*c*)**	9.16 (6.25, 12.06)	
**% infections due to hypnozoite reactivation.**	90%(86%, 92%)	
**Estimated treatment failure rate**	40% (32%, 48%)	

### Modeling primary infection and reactivation with *P*. *vivax*

A second difference between the Thai and PNG studies is the age of the cohorts, which was young in PNG, but included all ages in Thailand. Therefore we asked whether age may affect the proportion of infections arising from hypnozoite reactivation. Primary infection arises soon after infectious inoculation, and thus should be proportional to the current rate of infectious inoculation. By contrast, reactivation from hypnozoites requires first the establishment of a ‘reservoir’ of hypnozoites from previous infections, and then their later reactivation. Thus, for example, after the first exposure in life, only hynozoites laid down by the first inoculation can reactivate. However, after many exposures, reactivation can occur from hynozoites laid down at different times in the past. This is evident from previous studies of *P*. *vivax* clonotypes in infection. These studies have analysed the relationship between *P*. *vivax* clonotypes from baseline infection, and in subsequent infectious episodes. In adults, these clonotypes are very often unrelated, consistent with reactivation of hypnozoites laid down prior to the most recent infection event [13, 14, 22]. However, in children it is more likely that baseline infection and subsequent reactivation will be due to the same clonotype [23].

**Fig 5:**
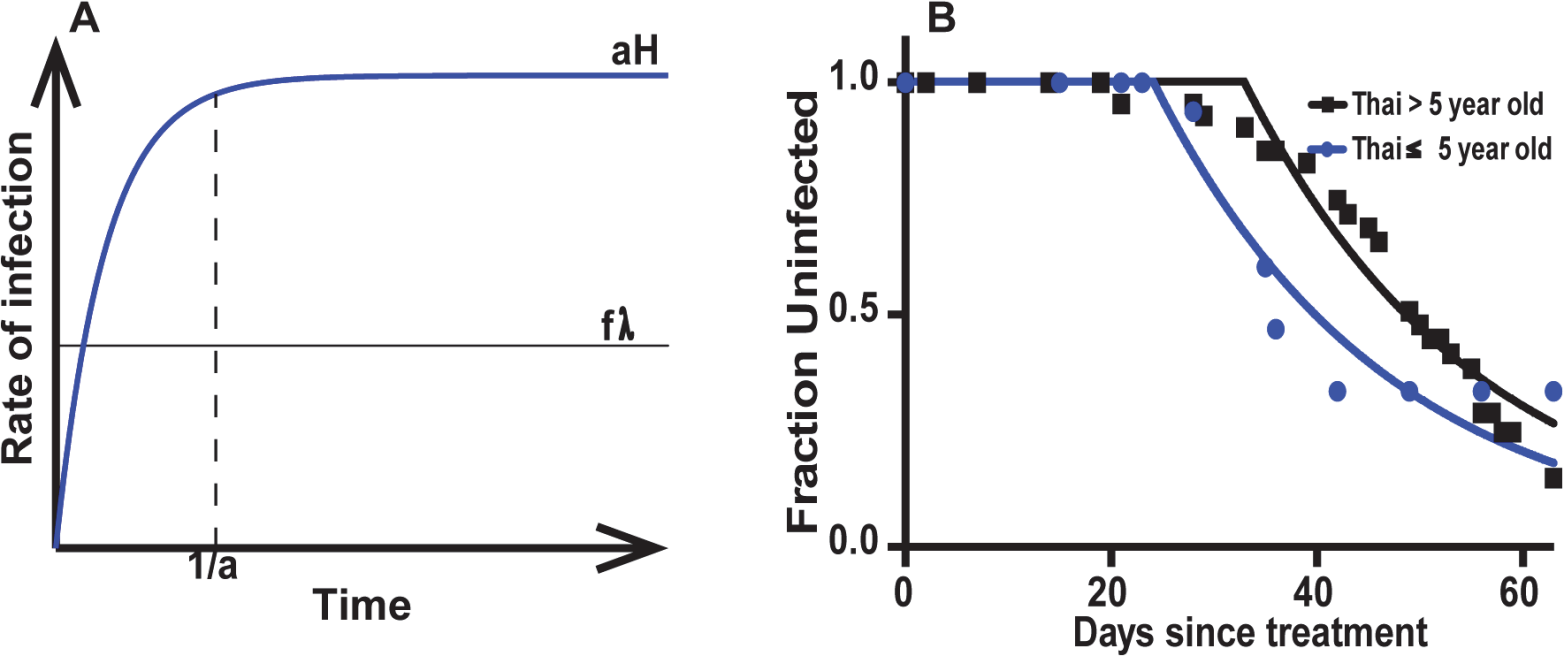
Dynamics of *P*. *vivax* infection with time. (A) Modeling the proportion of infections from new primary infection versus hypnozoite reactivation with time. In the presence of a constant rate of inoculation, the rate of primary infection (black line) will rapidly reach equilibrium and be constant with time. However, the rate of hypnozoite reactivation (blue line) will take some time to reach its steady state level. The time taken to reach this is dependent on the rate of reactivation of hypnozoites (*a*_*H*_). (B) shows best-fits of time to *Pv* infection in children < 5 years or subjects >5 years old in the Thai study.

We modeled the rates of primary infection and reactivation with age, to investigate how this might impact our analysis (using Equations (5), (6) and (7). The results suggest that for a given infection rate, the rate of primary infection is constant over time, but the rate of reactivation takes some time to reach its long-term level. Therefore at young ages we would expect an overall lower rate of infection (due to a lower rate of hypnozoite reactivation), as well as a higher ratio of primary infection to reactivation. How long this effect would be observed is directly related to the average time between laying down of hypnozoites and their subsequent reactivation (as shown in Fig. 5A). However, since the average time for hypnozoites to reactivate is thought to be of the order of months in tropical regions, this effect should only be present very early after initial exposure, and seems unlikely to have been the cause of the observed differences between the PNG and Thai cohorts.

The analysis above suggest that if age plays an important role in the ratio of primary infection to reactivation, then younger children treated with CQ in the Thai cohort should also exhibit an overall lower infection rate than older individuals (because of the reduced rate of hypnozoite reactivation). To test this we analysed the rate of infection of the children aged <5 years in the CQ treated cohort from the Thai study. Our fitting using Equation (2) showed no evidence for a significant difference in the rate of infection between those aged < 5 years or >5 years old in the Thai cohort (*p* = 0.2493, F-test, Fig. 5B). Thus, it seems unlikely that age alone would explain the difference between the Thai and PNG studies.

The model above considers how the infection rate from new inoculation and reactivation of *P*. *vivax* hypnozoites evolves with age in children exposed to infection. The same dynamics of accumulation of hypnozoites will also occur after successful primaquine therapy, when again the individual starts with no hypnozoite reservoir. After successful primaquine therapy both the number of hypnozoites and the rate of infection from hypnozoites will similarly increase over time.

### Understanding the effects of infection rate and seasonality

Other mechanisms for the differences between the Thai and PNG studies include the overall rate of inoculation, as well as seasonal fluctuations in this. Changes in the inoculation rate *per se* should not affect the ratio of primary infection to reactivation. That is, the average ratio of primary infection to reactivation is determined by the proportion of sporozoites progressing immediately to infection versus becoming hypnozoites, and is relatively independent of the rate of inoculation. Moreover, comparing the groups with treatment for blood stage infection only (CQ in Thailand and AS in PNG), the rate of infection in these groups was similar (0.043 vs. 0.034 infections per day, respectively). Once we accounted for treatment resistance, our estimated rate of new primary infection was also similar (0.0017 vs. 0.0032 per day, respectively). Thus, it seems unlikely that differences in the rate of infection were a major factor.

**Fig 6:**
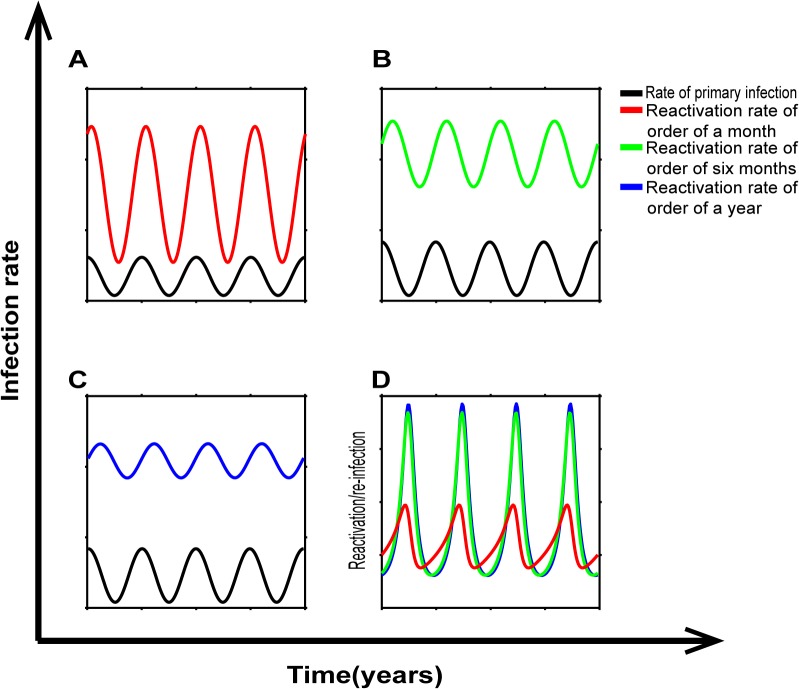
Impact of seasonality in transmission. (A–C) The rate of primary infection over time (black line) and hypnozoite reactivation with time (coloured lines) with seasonal transmission is plotted for different rates of hypnozoite reactivation. Rapid hypnozoite reactivation (half-life of one month) leads to rapid fluctuation in the rate of infection from hypnozoite reactivation. Slower rates of reactivation, equivalent of a half-life of six months (B) or 12 months (C) lead to more stable levels of hypnozoite reactivation. (D), The effect of seasonality on the ratio of hypnozoite reactivation versus primary infection when reactivation is of order of a month (red line), order of six months (green line) and order of a year (blue line). The ratio of infections from hypnozoite reactivation versus primary infection is paradoxically more stable in the setting of rapid hypnozoite reactivation (red line).

Seasonal fluctuations in the rate of new infections will have a direct effect on the rate of primary infection over time. However, if the reactivation of hypnozoites happens on a longer timescale, it may be less susceptible to seasonal variation, and thus alter the ratio of primary infection to hypnozoite reactivation over the seasons. Since the PNG study may have a higher degree of seasonality compared with the Thai study [8, 9], we explored the predicted impact of seasonality. We modeled a sinusoidal variation in the rate of overall infection (Equation (9)), and a constant fraction of infections becoming primary infection (Equation (5) or being laid down as hypnozoites (Equation (6)) and later reactivating (Equation (7)). The rate of infection from primary infection and hypnozoite reactivation thus evolve over time as described in Equations (5) and (8) respectively. When the average time to hypnozoite reactivation was short (one month, Fig. 6A), the number of hypnozoites and rate of hypnozoite reactivation fluctuates a lot over time, mirroring (with slight delay) the fluctuations in primary infection. However, as the average time to hypnozoite reactivation gets longer (Fig. 6B and 6C)), the number of hypnozoites and rate of hypnozoite reactivation becomes less variable with season. For our purposes, we are most interested in how seasonal fluctuation in infection rate might affect the ratio of primary infection to hypnozoite reactivation. Somewhat counter-intuitively, the shorter the time to hypnozoite reactivation, the less seasonal fluctuation in this ratio is seen (Fig. 6D). Thus, seasonality of infection may significantly alter the ratio, but this is least likely to have an effect with tropical strains of *P*. *vivax*, where the time to hypnozoite reactivation is thought to be relatively short. In our modeling, the rate of hypnozoite reactivation was assumed to be independent of season. However some have suggested that there may be a seasonality in presentation from UK residents returning from endemic regions[24], supporting a varying reactivation rate with season, which could further affect the ratio of primary infections to reactivation.

## Discussion

In the study we have estimated the relative contribution of new primary infection and the reactivation of dormant liver-stage hypnozoites to the rate of blood stage infection in individuals living in the *P*. *vivax* endemic areas. Our modeling results showed that the vast majority of infections (96%) in Thailand were due to hypnozoite reactivation. The proportion of infection due to hypnozoite reactivation in PNG was less clear. Considering only the early phase of infection and 100% efficacy of primaquine, we would estimate that only 70% of infections in PNG were due to hypnozoite reactivation. However, we found that when the full time course of infection was considered, we had a significantly better fit to a model in which up to 40% of PNG individuals were resistant to primaquine therapy. If this level of primaquine resistance in the population is correct, then 90% of the infections were due to hypnozoite reactivation in the PNG cohort study.

A number of factors differed between the Thai and PNG studies that might affect our results. Firstly, the age of the cohorts was different, with the PNG cohort focused on children 1–5 years of age, whereas the Thai cohort included individuals of all ages. Age may play a role in susceptibility to *P*. *vivax* infection [25, 26], and in the ratio of primary infection to reactivation. However, if we restricted our analysis to the 1–5 years age group in the Thai study, we found a similar overall infection rate to the rest of the cohort. Secondly, there may have been differences in the infection rate or seasonality of infection between the studies. However, again we found that these were unlikely to have a profound effect on the ratio of primary infection to reactivation. Since the drug dosage differed between PNG and Thailand, this may have contributed to treatment failure. However, since the dose of primaquine given was higher in the PNG study (0.5 mg/kg in PNG vs 0.25 mg/kg in Thailand), this would not appear to support less effective treatment in PNG. Finally, it has been suggested that *P*. *falciparum* infection may precipitate *P*. *vivax* hypnozoite reactivation [27, 28]. In the Thai cohort, patients who had mixed infection at enrolment were excluded. However, in the PNG cohort these patients were included. Thus, if recent infection with *P*. *falciparum* precipitates *P*. *vivax* reactivation, this may have driven earlier reactivation in some patients the PNG study. As we do not have data on which patients were co-infected at enrolment in this cohort, we are unable to exclude this as a factor in our study.

In our analysis of primary infection and reactivation, we first assumed a constant ‘force of infection’. That is, we assumed that the rate of infectious inoculation, new primary infection, and reactivation of hypnozoites was relatively constant over the period of study. While the assumption of stable infection rate is easy to understand, the assumption of a constant rate of reactivation is less clear. That is, one might expect that following a single infection event, hypnozoites may be more likely to activate early than late, and therefore we might see a decrease in hypnozoite reactivation rate over time [19]. However, we show that since individuals are presumed exposed to the same rate of inoculation before and after therapy, they will be in a ‘steady state’ in which, regardless of the distribution of times for hypnozoites to reactivate, the observed rate of infection from hypnozoite reactivation is effectively constant with time. Exploring the impact of seasonal variations in infection rate, we find that these can significantly affect the ratio of primary infection to reactivation over time.

Our studies indicated that the infection dynamics of the AS + PQ group in PNG could be best fit by assuming that primaquine was ineffective in a proportion of individuals in the PNG study. This not only provided a significantly better fit to the shape of the AS+PQ reinfection curves, but also led to a ratio of primary infection too reactivation that was much more similar to the Thai study. Here, it is important to differentiate parasite resistance to primaquine from ineffectiveness of primaquine in a given host. The former would result in the clearance of only a proportion of [susceptible] hypnozoites, whereas the latter would lead to clearance of all hypnozoites in a proportion of individuals. The possibility of true primaquine resistance is questioned by some, who suggest that variation in host genetics may lead to ineffectiveness of primaquine in a subset of individuals [29]. Primaquine is not itself active, and requires metabolism by cytochrome P450 isoenzymes to its active form [30]. Human populations vary in the proportion of functional and non-functional CYP2D6 alleles, with PNG having a high proportion of novel and uncharacterized alleles [31]. Although these novel alleles have not been biochemically characterized, many are assumed to be active [32]. In addition, there appears evidence that parasites acquired in PNG are less susceptible to primaquine. Thus, it seems highly likely that treatment failure is indeed higher in PNG.

Understanding the relative contribution of primary infection and relapse in *P*. *vivax* infection is important to driving future treatment strategies. We have developed a novel analytical framework that allows estimation of the primary infection to reactivation ratio. This work indicates that reactivation of hypnozoites contributes 90–96% of observed *P*. *vivax* infections. In addition, our work suggests approaches for understanding the level of primaquine resistance in different populations. Understanding the mechanism of hypnozoite reactivation and identifying optimal approaches for targeting the hypnozoite reservoir will greatly reduce the burden of *P*. *vivax* infection.
